# Peritumoral Clefts in Basal Cell Carcinoma: Matrix Metabolism and Primary Cilium

**DOI:** 10.7759/cureus.58316

**Published:** 2024-04-15

**Authors:** Jesús Iván Martínez-Ortega

**Affiliations:** 1 Dermatology, Instituto Dermatológico de Jalisco, Zapopan, MEX; 2 Histology, Autonomous University of Nuevo León, Monterrey, MEX

**Keywords:** peritumoral cleft, extracellular matrix, primary cilia, tissue retraction, basal cell carcinoma

## Abstract

The phenomenon of tissue retraction, characterized by peritumoral spaces or clefts, is prominent in basal cell carcinoma (BCC) tumors, yet its underlying mechanisms remain unclear. Proposed factors include changes in cell structures, enzymatic activity, and alterations in the Hedgehog (Hh) signaling pathway. This article discusses these factors and proposes that structural changes in BCC cells' primary cilia may contribute to matrix alterations, leading to the formation of peritumoral clefts. Further research is needed to confirm these hypotheses and understand BCC's unique growth patterns.

## Editorial

The peculiar phenomenon of "tissue retraction," appearing as spaces, clefts, or peritumoral lacunae, has been noted since the early histopathological examinations dating back to 1903 by Krompecher [1]. The appearance of these peritumoral clefts or palisading features surrounding the characteristic basaloid tumor cells has intrigued researchers and pathologists for years. While the simplest explanation may be attributed to tissue retraction or artifacts [1,2], it persists even under in vivo imaging [2]. Additionally, a persistent question remains: why does this distinctive characteristic prominently manifest in basal cell carcinoma (BCC) tumors and rarely in other scenarios?"

Various theories have been proposed regarding the formation of spaces around BCC tumors, including changes in desmosomal structures leading to detachment of the basal membrane [1] or disruption by enzymes [2], or even laminar detachment of peripheral tumoral cells [1]. However, while these theories offer potential explanations, they have not been conclusive.

Others have noted mucin and glycoprotein deposits in the clefts using special stains [2]. Moreover, it has shown an elevated synthesis of hyaluronic acid in the clefts [3]. Additionally, recent morphometric analyses suggest that enzymatic activity, particularly the degradation of hyaluronic acid and glycosaminoglycans by tumor enzymes, leads to cleft formation. Fibroblasts in the stroma contract the collagenous matrix, contributing to this phenomenon. Unlike fixation artifacts, peritumoral clefting is seen as a visible outcome of matrix synthesis or degradation associated with the tumor's enzymatic activity and stromal response [2,3].

BCC is characterized by the aberrant activation of the Sonic Hedgehog (Hh) signaling pathway, which relies on the primary cilium. Moreover, the upregulation of the ciliogenic program in BCC cells frequently results in the presence of multiple primary cilia [4]. These structural changes, coupled with the functional hyperactivation of the Hh chemical signal, may impact various, previously unsuspected functions of the primary cilium.

Collins and Wann hypothesized that the primary cilium is responsible for the expression, secretion, and proteolytic remodeling of the extracellular matrix. This hypothesis is based on numerous studies and accumulating evidence, although none of the evidence directly and experimentally proves it [5].

**Figure 1:**
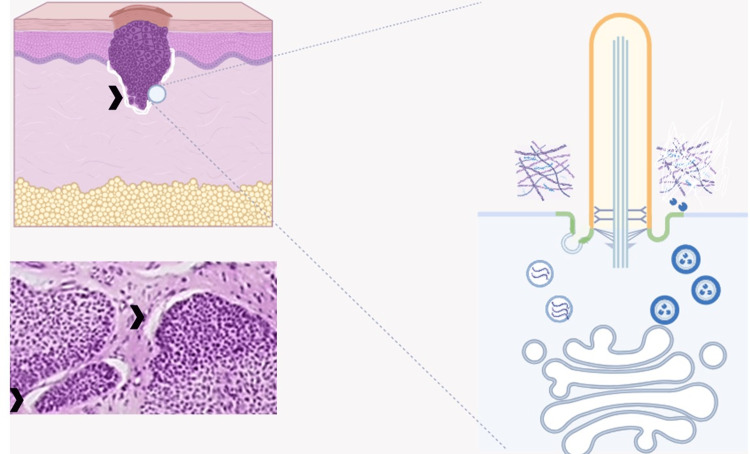
Peritumoral Cleft Formation, Matrix Metabolism, and Primary Cilium On the left: basal cell carcinoma (BCC) with peritumoral cleft (arrowhead); below, a real photographic representation of a pathological peritumoral cleft (arrowhead). On the right: the proposed involvement of an altered primary cilium in matrix synthesis and degradation, presumably related to cleft formation. Created with BioRender.

Altogether, I posit that structural alterations in the primary cilium of BCC cells may instigate functional modifications in matrix degradation and synthesis, potentially contributing to the development of peritumoral clefts. Experimental validation is essential to confirm the primary cilium's role in extracellular matrix homeostasis (Figure 1).

Specifically, further investigations should examine whether abnormal signaling and/or structural changes in the primary cilium of BCC cells contribute to alterations in matrix degradation, ultimately leading to cleft formation. Furthermore, investigating whether these alterations in the extracellular matrix physically restrict the tumor, contributing to its distinctive slow growth and infrequent dissemination observed in BCC, would enrich our understanding of this skin cancer's unique features.
